# Anticancer Drug Discovery Based on Natural Products: From Computational Approaches to Clinical Studies

**DOI:** 10.3390/biomedicines12010201

**Published:** 2024-01-16

**Authors:** Pritee Chunarkar-Patil, Mohammed Kaleem, Richa Mishra, Subhasree Ray, Aftab Ahmad, Devvret Verma, Sagar Bhayye, Rajni Dubey, Himanshu Narayan Singh, Sanjay Kumar

**Affiliations:** 1 Department of Bioinformatics, Rajiv Gandhi Institute of IT and Biotechnology, Bharati Vidyapeeth (Deemed to be University), Pune 411046, Maharashtra, India; 2 Department of Pharmacology, Dadasaheb Balpande, College of Pharmacy, Nagpur 440037, Maharashtra, India; kaleemmubin88@gmail.com; 3 Department of Computer Engineering, Parul University, Ta. Waghodia, Vadodara 391760, Gujarat, India; richa.mishra31240@paruluniversity.ac.in; 4 Department of Life Science, Sharda School of Basic Sciences and Research, Greater Noida 201310, Uttar Pradesh, India; 5 Health Information Technology Department, The Applied College, King Abdulaziz University, Jeddah 21589, Saudi Arabia; 6 Pharmacovigilance and Medication Safety Unit, Center of Research Excellence for Drug Research and Pharmaceutical Industries, King Abdulaziz University, Jeddah 21589, Saudi Arabia; 7 Department of Biotechnology, Graphic Era (Deemed to be University), Dehradun 248002, Uttarkhand, India; devvret@gmail.com; 8 Division of Cardiology, Department of Internal Medicine, Taipei Medical University Hospital, Taipei 11031, Taiwan; 9 Department of Systems Biology, Columbia University Irving Medical Center, New York, NY 10032, USA; 10 Biological and Bio-Computational Lab, Department of Life Science, Sharda School of Basic Sciences and Research, Sharda University, Greater Noida 201310, Uttar Pradesh, India

**Keywords:** natural product, anticancer drug discovery, computational drug design, clinical trials, molecular dynamics, drug design

## Abstract

Globally, malignancies cause one out of six mortalities, which is a serious health problem. Cancer therapy has always been challenging, apart from major advances in immunotherapies, stem cell transplantation, targeted therapies, hormonal therapies, precision medicine, and palliative care, and traditional therapies such as surgery, radiation therapy, and chemotherapy. Natural products are integral to the development of innovative anticancer drugs in cancer research, offering the scientific community the possibility of exploring novel natural compounds against cancers. The role of natural products like Vincristine and Vinblastine has been thoroughly implicated in the management of leukemia and Hodgkin’s disease. The computational method is the initial key approach in drug discovery, among various approaches. This review investigates the synergy between natural products and computational techniques, and highlights their significance in the drug discovery process. The transition from computational to experimental validation has been highlighted through in vitro and in vivo studies, with examples such as betulinic acid and withaferin A. The path toward therapeutic applications have been demonstrated through clinical studies of compounds such as silvestrol and artemisinin, from preclinical investigations to clinical trials. This article also addresses the challenges and limitations in the development of natural products as potential anti-cancer drugs. Moreover, the integration of deep learning and artificial intelligence with traditional computational drug discovery methods may be useful for enhancing the anticancer potential of natural products.

## 1. Introduction

Cancer is a group of diseases characterized by uncontrolled growth and spread of abnormal cells in the body [1,2,3]. Genetic and epigenetic alterations lead to the switch-on and switch-off of tumor suppressor genes (*TSGs*) and oncogenes [2,4]. These abnormal cells can form tumors and invade nearby tissues and organs, thereby interfering with normal body functions. There are more than 100 different types of cancers, each with its own characteristics, risk factors, and treatment options. In 2020, approximately 18.1 million new cases of cancer were reported worldwide, excluding non-melanoma skin cancer, with 8.8 million (48%) in females and 9.3 million (52%) in males. This resulted in a ratio of 10 males to every 9.5 females. The global age-standardized incidence rate was 178.1 per 100,000 females and 206.9 per 100,000 males. The four most common types of cancer worldwide are breast, lung, bowel (including anus), and prostate cancers, which collectively account for 43% of all new cases [5]. In 2023, 1,958,310 new cancer cases and 609,820 cancer-related deaths are projected to occur in the US. The incidence of prostate cancer increased by 3% annually from 2014 to 2019, resulting in 99,000 additional cases. Cancer is caused by the accumulation of genetic mutations over time [5]. These mutations can result from various factors, including exposure to carcinogens (cancer-causing substances), genetic predisposition, lifestyle choices (such as smoking and diet), infections, and other environmental factors [6,7]. Cancers can be broadly categorized into two main types: benign and malignant. Benign tumors are noncancerous and do not spread to other parts of the body. In addition to invading the adjacent tissues, malignant tumors can metastasize (proliferate) to other anatomical sites. The spread of tumor cells frequently determines the degree of disease, which is known as staging. This offers medical personnel prognostic information and aids in therapeutic decision-making. The most frequently employed staging approach to classify different forms of cancer is the TNM system, an abbreviation for tumor size, lymph node involvement, and metastasis [8].

Leukemia, breast cancer, lung cancer, prostate cancer, colorectal cancer, and skin cancer (melanoma) are among the most prevalent forms of cancer [9]. Different types possess distinct properties, which may necessitate distinct treatment methodologies. Frequently, cancer risk can be reduced by adopting healthy lifestyles. These include refraining from smoking, adhering to a nutritious diet, engaging in consistent physical activity, shielding oneself from excessive solar radiation, and preventing contact with recognized carcinogens. Mammograms, Pap smears, colonoscopies, and prostate-specific antigen (PSA) tests are examples of screenings that can aid in the early, curable detection of cancer [10].

**Figure 1:**
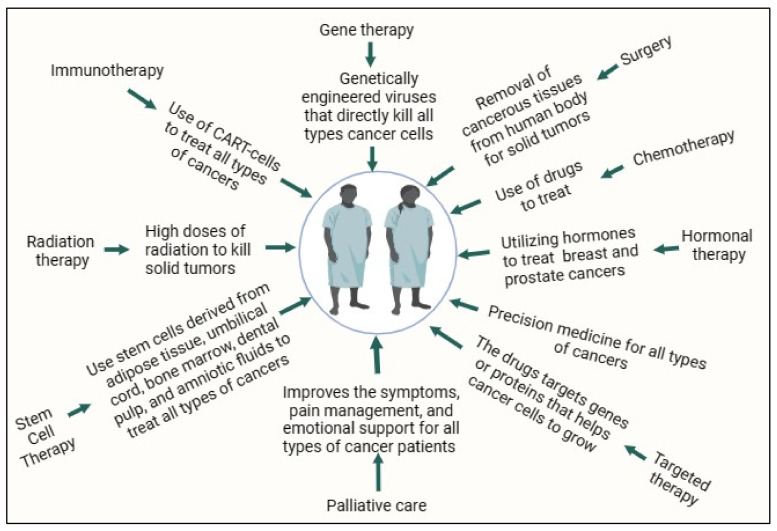
Various therapeutic strategies for cancer treatment.

Moreover, early identification of cancer frequently results in improved treatment outcomes. The treatment options for cancer differ, according to the nature and stage of the disease. Stem cell transplantation, radiation therapy, chemotherapy, targeted therapy, immunotherapy, and hormone therapy are the most common therapeutic techniques [11]. Possible treatment modalities include a combination of these methods. Prolonged investigations pertaining to the etiology, prevention, and therapeutic aspects of cancer have recently yielded noteworthy progress. These include the advancement of precision medical strategies, targeted medicines, and immunotherapies [12]. The prognosis of many cancer patients has substantially improved as a result of ongoing research and advancements in medical therapies, despite the fact that cancer continues to be a major worldwide health concern. Advancements in cancer therapy, early detection, and prevention are critical components of the ongoing battle against this disease. Oncology therapy, or cancer therapy, encompasses a range of medical procedures and therapies implemented to control and eradicate cancer [13]. Cancer type and stage, in addition to the patient’s general health and treatment objectives, influence treatment selection. Cancer therapy can be classified into several primary categories, on a large scale. Surgical techniques to remove malignant tumors and surrounding tissues comprise the first category [14]. Surgery is often the primary treatment for solid tumors such as breast cancer, lung cancer, and colon cancer. It can also be used for diagnostic purposes, staging, and alleviation of symptoms and complications [14,15]. The second type is radiation therapy, or radiotherapy, which uses high-energy beams of radiation to target and kill cancer cells [16,17]. It can be used as a monotherapy or in combination with other therapies. Radiation therapy is often employed for localized tumors, and may be administered before or after surgery. The third category is chemotherapy, which involves the use of drugs to destroy cancerous cells or inhibit their growth [18], which can be administered either orally or parentally. Chemotherapy is often used for systemic or metastatic cancers, but can also be part of adjuvant therapy following surgery. The fourth category is immunotherapy, or biological therapy, which harnesses the body’s immune system to recognize and attack cancer cells [19]. These include monoclonal antibodies, checkpoint inhibitors, cytokines, and cancer vaccines. Immunotherapy has shown remarkable improvement in treating various cancer types, including melanoma, lung cancer, and some types of leukemia. The fifth category is targeted therapy, which uses drugs that specifically target cancer cells by interfering with the molecules or pathways that drive their growth and spread [20]. These drugs are often more precise and have fewer side effects than traditional chemotherapies. Targeted therapies are used to treat tumors with specific genetic mutations or other targetable characteristics. The sixth type is associated with the use of hormones, which are primarily used for hormone-sensitive cancers, including breast and prostate cancer [21]. This involves medications that block the effects of hormones or reduce their production, as hormones can fuel the growth of certain cancers. The seventh category is known as stem cell transplantation (bone marrow transplantation), which is used to replace damaged or cancerous bone marrow with healthy stem cells [22]. It is often used in the treatment of blood-related cancers, including leukemia and lymphoma. The eighth type is precision medicine, which involves tailoring cancer treatment to an individual’s specific genetic and molecular profiles [23]. This approach aims to maximize the effectiveness of the therapy while minimizing side effects. The ninth type is palliative care, which is an indispensable component of cancer therapy that emphasizes improving the quality of life of patients with advanced or terminal cancer [24]. It addresses symptoms, pain management, and emotional support, and may be combined with curative treatments. Cancer therapy is also known as multimodal therapy, often administered as a combination of these treatments, which are summarized in Figure 1.

Computational modeling plays a crucial role in anticancer drug discovery, including the identification and development of anticancer compounds. Some of the computational methods are used to develop natural products as anticancer agents, which involves the following: molecular docking for virtual screening and binding site validation [25], pharmacophore modeling to identify pharmacophores [26], quantitative structure–activity relationship (QSAR) modeling to predict activity and toxicity [27], molecular dynamics simulation to understand binding mode, affinity, and solvent effect [28], ADME property prediction [29], network pharmacology to construct and analyze networks of protein–protein interactions and pathways affected by natural compounds [30], and machine learning and artificial intelligence algorithm-based modeling to predict various ADMET properties and optimize natural compounds [31]. Computational modeling can significantly accelerate the drug discovery process, making it more cost-effective and efficient. However, it is essential to validate the results of computational studies through experimental testing, to ensure the safety and efficacy of potential natural anticancer compounds. This multidisciplinary approach, which combines computational modeling with experimental validation, holds great promise for the development of new and effective cancer treatments.

## 2. Historical Perspective

Since ancient times, natural drugs have been central to medicinal practices and have served as primary healthcare solutions across various cultures. These drugs, encompassing plant-based, animal-based, and mineral-based medicines, have been particularly prominent in India and China, which are often considered mother nations for the utilization of natural-product drugs [32]. Approximately 80% of the world’s population relies on traditional medicinal systems, and plant-derived drugs have significant therapeutic value [33]. Medicinal plants, the key components of these systems, have been used for their healing properties for centuries.

According to Ashok and Devasagayam (2007), nearly 70% of Indians rely on natural medications, a figure that increases to 90% in Africa. Herbal drugs, a critical component of natural drugs, play a significant role in Ayurveda, yoga, Unani, Siddha, homeopathy, and naturopathy [34]. Particularly in the realm of cancer, the convergence of ethnobotany and traditional knowledge is crucial to the development and use of natural medicines. Ethnobotanical investigations, which focus on the utilization of plants and other natural compounds by many civilizations, have yielded vital knowledge regarding their potential therapeutic attributes. Scientists and researchers have frequently been influenced by this conventional approach when attempting to identify plants that may possess anticancer properties. The utilization of periwinkle in traditional medicine, for instance, has resulted in the identification of vinca alkaloids, which are essential chemotherapeutic agents. The persistent integration of conventional wisdom and contemporary scientific investigation remains a pivotal catalyst in the ongoing pursuit of novel anticancer substances [35,36,37].

In the realm of anticancer drug discovery, natural products are pivotal because they offer a diverse array of therapeutic possibilities. This historical overview traces the journey from traditional medicine to modern cancer therapy. Initially, plant- and animal-derived compounds were used in traditional medicine, laying the foundation for understanding their therapeutic potential [38,39]. The 20th century saw a significant shift in the systematic isolation and characterization of active compounds from natural sources, leading to breakthroughs such as Vinca alkaloids, which had a substantial impact on treating challenging cancer types [39,40,41].

Progression in extraction and separation methodologies has facilitated the transformation of natural substances from conventional applications to contemporary medicinal agents. Previously, techniques employed to extract therapeutic chemicals from plants and other natural sources were frequently primitive, and lacked efficiency. However, since the introduction of modern technologies and chemistry, these techniques have progressed considerably. The use of methodologies such as ultrasonic, microwave-assisted, and supercritical fluid extraction has facilitated the selective and effective separation of active chemicals [42,43]. In addition to increasing the yield and purity of natural products, these developments have enabled the identification of novel molecules with anticancer effects. The capacity to extract and analyze the distinct constituents of conventional treatments has proven crucial in comprehending their mechanisms of action and formulating standardized pharmacological drugs [44,45].

The development of Taxol, or paclitaxel, from the Pacific yew tree epitomizes this evolution, balancing ecological sustainability with therapeutic advancement. Discovered as part of the National Cancer Institute program in the 1960s, Taxol’s journey highlights the integration of emerging technologies such as high-throughput screening and computational modelling in drug discovery [38,46,47]. Despite challenges such as supply issues and ecological impacts, innovations in synthetic and semi-synthetic methodologies have paved the way for the next generation of anticancer drugs [48,49].

Taxol’s mechanism of action, promoting tubulin assembly into microtubules and stabilizing them against disassembly, is distinct from other treatments of its time, making it a unique and effective anticancer agent [50]. However, the development of Taxol faced challenges, owing to the low abundance of the compound in its natural source and the ecological implications of harvesting yew trees. Advances in semi-synthesis from more abundant yew species eventually led to FDA approval of ovarian cancer treatment in the early 1990s [49,51]. Taxol’s success story not only underlines the importance of natural products in drug discovery, but also emphasizes the need for sustainable sourcing and interdisciplinary collaboration in pharmaceutical development.

The incorporation of natural substances into contemporary pharmacopeia signifies a meaningful advancement in pharmaceutical exploration. Although the utilization of natural chemicals was first inspired by traditional medicine, their integration into conventional healthcare has necessitated stringent scientific verification and standardization. Extensive pharmacological and toxicological testing was conducted as part of this procedure, to guarantee safety and effectiveness in strict adherence to rigorous standards of regulatory bodies [40]. The incorporation of natural compounds, such as Taxol, into pharmacopeia represents a transition from anecdotal and empirical use to evidence-based medicine. Furthermore, it emphasized the potential of natural products as an abundant reservoir of innovative therapeutic agents with the capacity to tackle a multitude of complex health ailments, such as diverse forms of cancer [52,53].

## 3. The Importance and Potential of Natural Products in Drug Discovery

Promising and novel cancer therapies have been explored and studied in natural compounds and their structural analogs, and show exceptional variation in chemicals. In addition, the distinct molecular characteristics of natural products enable them to offer greater safety and effectiveness [47]. Chemotherapy drugs like doxorubicin and cisplatin, as well as radiotherapy, are commonly employed in cancer treatment, but are associated with severe adverse reactions and toxic side effects. Radiotherapy, in particular, can lead to cognitive dysfunction and a decline in brain function [54]. Additionally, chemotherapy may result in secondary tumors and damage to normal tissues, presenting challenges for cancer survivors. Common issues during chemotherapy include bone marrow suppression, causing immunosuppression, and various toxicities such as liver, kidney, and heart toxicity [55]. For instance, cisplatin can induce nausea, vomiting, acute kidney injury, neurotoxicity, and ototoxicity. Some chemotherapy drugs may not effectively target less-active cancer cells, influencing overall survival and prognosis, negatively [56].

In recent times, natural compounds have gained importance in cancer prevention and treatment. These compounds, including phenols (such as curcumin, quercetin, resveratrol, and capsaicin), flavonoids (quercetin, tanshensin IIa, and icariin), terpenoids (andrographolide, artesunate, and atractylodes), alkaloids (matrine, berberine, and piperine), and others, play a crucial role. They exhibit anti-inflammatory properties, promote cell apoptosis, inhibit invasion and metastasis, and enhance immune responses. These natural compounds have demonstrated efficacy against various cancers like lung cancer, breast cancer, and ovarian cancer [57].

Compounds generated from bacteria, plants, and marine organisms are considered to be natural products. Throughout history, natural products have been crucial for the advancement in cancer treatments. Research on anti-cancer medication employs natural products because of their extensive chemical diversity, distinctive structural characteristics, and biological activity, which has less toxicity. As some of these substances have undergone evolutionary adaptations to protect species from illnesses, including cancer, they are promising candidates for anticancer drugs. Screening the anti-cancer activity of natural-product extracts is a customary initial step taken by researchers. Subsequently, promising extracts are isolated and purified to determine their precise active components. It is frequently possible to enhance the quality of natural products by chemical modification, as in the case of improving their bioavailability or targeting particular types of cancer cells. By synthesizing analogs or derivatives, medicinal chemists can augment the drug-like characteristics of substances.

**Figure 2:**
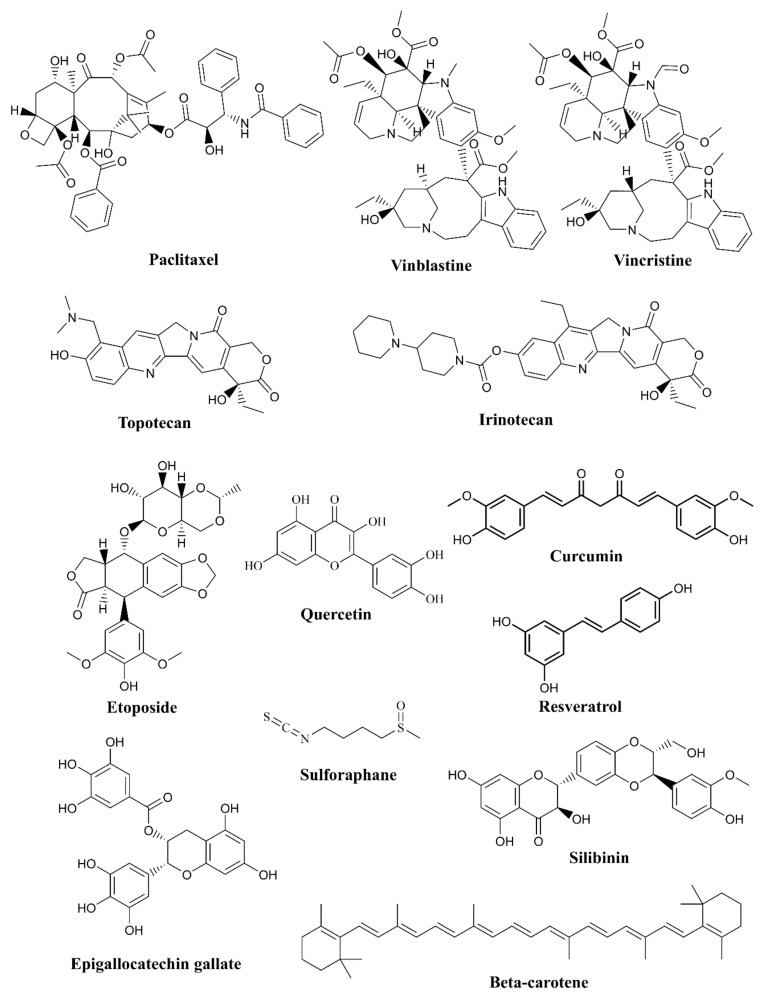
Natural compounds from various sources exhibit anti-cancer activity.

About fifty percent of drugs originated from natural substances. These could be compounds that are either semi-synthetic or obtained from flora [58]. Examples of natural products used in anti-cancer drug development include the following (Figure 2 and Table 1).

1. Paclitaxel: derived from the Pacific yew tree, and is used to treat various cancers, including breast, ovarian, and lung cancers [59].
2. Vinblastine and Vincristine: these alkaloids are derived from the *Madagascar periwinkle* plant and are used in the treatment of leukemia and lymphoma [60].
3. Camptothecin: originally isolated from the Chinese tree *Camptotheca acuminata*, derivatives of this compound, such as Topotecan and Irinotecan, are used in the treatment of ovarian and colorectal cancers [61,62].
4. Etoposide: derived from the mayapple plant, etoposide is used to treat lung and testicular cancer [63].

**Figure 3:**
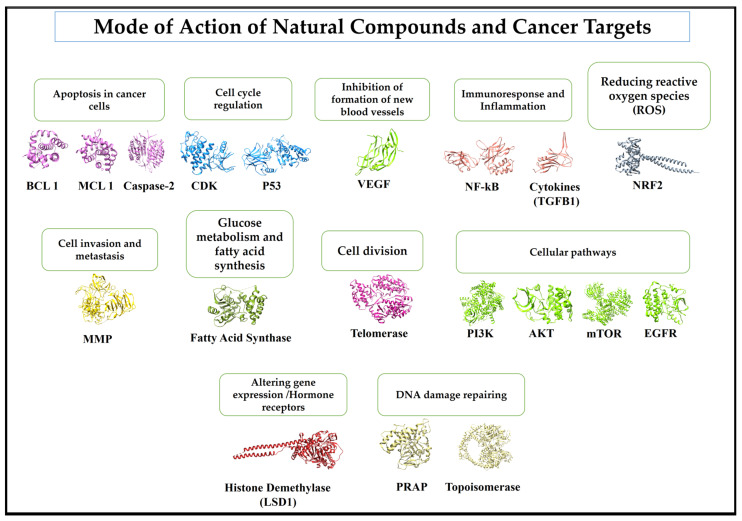
Mode of action and molecular targets of natural compounds involved in cancer development. The molecular targets are BCL-1, MCL-1, Caspase-2, CDK, p53, VEGF (Vesicular Endothelial Growth Factor), NF-kB, TGFB1, NRF2, MMP, Fatty Acid Synthase, Tellomarase, P13, AKT, mTOR, EGFR, Histone Demthylase, PRAP, and Topoisomease.

Natural products have several advantages in drug discovery, including structural diversity, potential for multi-target effects, and the presence of compounds that can overcome drug resistance. However, the challenges in sourcing and standardizing natural products, as well as issues related to patent protection, can complicate their development as anticancer drugs. Nevertheless, ongoing research in this field continues to yield promising results for novel cancer treatments. These compounds can interact with various molecular pathways and cellular processes associated with cancer development and progression (Figure 3). The common mode of action for some cancer targets are like the following: *Bcl-2*, *Bcl-xL*, and *Mcl-1*, and the activation of caspases enzymes to induce apoptosis in cancer cells [64]; inhibiting the cell cycle regulating enzymes/proteins such as cyclin-dependent kinases (CDKs) and p53 [65]; inhibiting the formation of new blood vessels in tumor cells by inhibiting enzyme-like vascular endothelial growth factor (VEGF) [66], by targeting proteins associated with immunoresponse and inflammation such as nuclear factor-κB (NF-κB) and cytokines [67]; reducing reactive oxygen species (ROS) and increasing the anti-oxidation defense against cancer-spreading cells [68]; inhibiting cancer cell invasion and metastasis by inhibiting group of enzymes known as matrix metalloproteinases (MMPs) and increasing the activity of Tissue Inhibitors of Metalloproteinases (TIMPs), which counter the effects of MMPs [69]; inhibition of DNA-damage-repairing enzyme such as poly(ADP-ribose) polymerase (PARP) [70]; altering gene expression in cancer cells by modifying DNA methylation and histone acetylation involved in epigenetics [71], in breast- and prostate-cancer natural-product target hormone receptors like estrogen and androgen receptors [72]; by interfering in cellular pathways such as PI3K/Akt/mTOR and the EGFR (Epidermal Growth Factor Receptor) pathway [73,74,75]; by inhibiting telomerase, an enzyme which helps to divide tumor cells indefinitely [76]; by inhibiting glucose metabolism and fatty acid synthesis [77,78]; and by inhibiting the topoisomerase group of enzymes to maintain damaged-DNA repair and replication [79,80].

**Table 1:** Various natural compounds used as anti-cancer treatments in treating various cancers via different pathways.

Natural Compound	Source	Mechanism of Action	Target Genes	Cancer	Reference
Curcumin	Turmeric (*Curcuma longa*)	Inhibits cell proliferation, induces apoptosis.	TNF, IL-1, VEGF, EGF, FGF, EGFR, HER-2, AR, NF-κB, AP-1, STAT	Breast, lung, skin, gastrointestinal, colorectal, prostate, head and neck.	[81]
Resveratrol	Grapes, berries, peanuts	Antioxidant, affects cell cycle regulation.	APE1/Ref-1, NF-κB, LSD1, MCP-1	Breast, cervical, uterine, blood, kidney, liver, eye, bladder, thyroid, esophageal, prostate, brain, lung, skin, gastric, colon, head and neck, bone, ovarian, and cervical.	[82]
Paclitaxel (Taxol)	Pacific yew tree (*Taxus brevifolia*)	Disrupts microtubule function.	AP-1, JNK1, p38, ERK1, IL-1α, IL-1β, TNF-α	Breast, ovarian, lung cancers.	[83]
Epigallocatechin gallate (EGCG)	*Green tea*	Antioxidant, induces apoptosis, inhibits proliferation.	retinoic acid receptor β (RARβ), CDH1 (e-cadherine gene), DAPK1, DNMT1, DNMT3B, HDAC1	Breast, lung, bladder, head and neck, prostate, colorectal.	[84]
Sulforaphane	Cruciferous vegetables	Induces detoxification enzymes, pro-apoptotic.	TIMP1, AURKA, CEP55, CRYAB, PLCE1, and MMP28, CRC	Colorectal.	[85]
Genistein	Soybeans	Inhibits angiogenesis, modulates hormone activity.	p21-WAF1, p16-INK4a, p21-WAF1 and p16-INK4a	Breast, colorectal, lung, pancreatic.	[86]
Quercetin	Apples, onions, tea, red wine	Antioxidant, anti-inflammatory, inhibits proliferation.	bcl-2-associated X protein (BAX), Cytochrome c release, Cysteine-aspartic proteases (caspase)-3, Caspase-9, Transforming growth factor β (TGF-β), Anti-apoptotic Bcl-2	Breast, prostate, colorectal, lung.	[87]
Capsaicin	Chili peppers	Induces apoptosis, inhibits cell growth.	c-myc, c-Ha-ras, p53	Breast, lung, bladder, colon and pancreatic, colorectal.	[88]
Silymarin (Silibinin)	*Milk thistle*	Antioxidant, anti-inflammatory, cell regeneration.	NF-кB, TGF-β, TNF-α, interferon-gamma, IL-2, IL-4, and COX-2	Breast, lung, colorectal, skin, pancreatic, prostate, gastrointestinal.	[89]
Berberine	*Berberis plants*	Inhibits cell progression, promotes apoptosis.	IL-1, TNF, IL-6, cyclooxygenase 2 and prostaglandin E2	Colon.	[90]
Ellagic acid	*Pomegranates*, *berries*, *nuts*	Antioxidant, anti-proliferative.	p53-dependent genes, NF-kB p50, p65, and the PPAR family	Colorectal, prostate, lung, bladder, ovarian, breast.	[91]
Lycopene	*Tomatoes*, *watermelon*, *pink grapefruit*	Antioxidant, anti-proliferative.	*IGFBP-3*, *c-fos*, and *uPAR*	Breast, colorectal, lung, pancreatic, ovarian, cervical.	[92]
Indole-3-carbinol	Cruciferous vegetables	Modulates estrogen metabolism, apoptosis.	CYP1A1, CYP1B1 and AhR	Lung, head and neck, bladder, breast.	[93]
Beta-glucans	Oats, barley, mushrooms	Stimulates immune response.	TLR-2/6, CR3	Breast, colorectal, prostate, ovarian.	[94]
Allicin	Garlic	Antioxidant, anti-proliferative, pro-apoptotic.	E2F1, E2F2, and E2F3	Breast, bladder, lung, colorectal, prostate.	[95]
Catechins	Tea, cocoa, fruits	Antioxidant, anti-inflammatory, anti-proliferative.	JNK, MAP kinase, JAKs, BCL-2, and Nrf2	Colorectal, pancreatic, lung, breast.	[96]
Ursolic acid	Apples, basil, cranberries	Inhibits metastasis, induces apoptosis.	MMP-9, CT45A2, Bcl-2, Bcl-xL, and BAX	Breast.	[97]
Limonene	Citrus peels	Induces detoxification enzymes, anti-proliferative.	Bcl-2-associated X protein (BAX), Cytochrome c release, Cysteine-aspartic proteases (caspase)-3, Caspase-9, Transforming growth factor β (TGF-β), Anti-apoptotic Bcl-2	These are not directly associated with causing specific cancers, but rather are involved in cellular pathways related to apoptosis (programmed cell death) and regulation of cell survival.	[98]
Vinblastine	Periwinkle plant (*Catharanthus roseus*)	Inhibits microtubule assembly.	*CCNB1 and AURKA*	Breast, colorectal, lung, ovarian, prostate.	[99]
Vincristine	Periwinkle plant (*Catharanthus roseus*)	Binds to tubulin, inhibits microtubule formation.	CYP3A4, CYP3A5	Liver.	[60]
Topotecan	Happy tree (*Camptotheca acuminata*)	Inhibits DNA topoisomerase I.	ABCB1, ABCG2, ALDH1A1, IFIH1, SAMD4 and EPHA3	Breast, ovarian, colon.	[100]
Irinotecan	Happy tree (*Camptotheca acuminata*)	Inhibits DNA topoisomerase I.	UGT1A1	It is not directly responsible for causing cance; variations in this gene can influence how the body processes certain chemotherapy drugs used in cancer treatment.	[101]
Etoposide	Mayapple plant (*Podophyllum peltatum*)	Inhibits DNA topoisomerase II.	SEMA5A, SLC7A6 and PRMT7	For these genes, ongoing research might reveal their specific associations with certain cancers or their roles in cancer biology. The understanding of their involvement in cancer development and progression might evolve as more studies uncover their molecular mechanisms and connections to different cancer types.	[102]
Beta-carotene	*Carrots*, *sweet potatoes*, *spinach*	Antioxidant, modulates immune response.	CD38, NCF1B, and ITGAL	These genes are involved in various biological processes, including immune response and cell signalling. Their associations with specific cancers are not as prominent as some other genes, but they have been implicated in certain contexts.	[103]

## 4. Present Status of Natural Compounds

Many natural compounds are under investigation in preclinical and clinical trials for their potential use in various medical applications, including cancer treatment, anti-inflammatory agents, and antioxidants (Figure 2). These compounds are derived from plants, marine organisms, and other microorganisms. Some examples of natural compounds that have undergone, or are currently undergoing, pre-clinical and clinical trials are Curcumin, which was tested for anti-inflammatory, antioxidant, and potential anti-cancer properties [104]; Resveratrol, which has been tested in both preclinical and clinical trials for cardioprotective and anti-aging effects [105]; Epigallocatechin Gallate (EGCG), which is being investigated for the treatment of various diseases, including cancer, neurodegenerative disorders, and metabolic syndrome [106]; Paclitaxel, which is an established chemotherapy drug for various cancers, including breast, ovarian, and lung cancer [107]; Quercetin, which has shown promising results in pre-clinical as well as clinical trials as an anti-inflammatory, antioxidant, and potential anti-cancer agent [108]; and Camptothecin and its derivatives, such as Topotecan and Irinotecan, which are used in the treatment of ovarian and colorectal cancer [109]. Clinical trials of Beta-carotene have explored its potential benefits, such as reducing the risk of certain cancers and eye conditions [110]. Sulforaphane is under study in pre-clinical and clinical trials for its potential anti-cancer and antioxidant effects [111]. Silibinin has shown potential in preclinical and early-stage clinical trials for various cancers and liver diseases, including hepatitis and cirrhosis [112]. These natural compounds are just a few examples of substances from nature that are being investigated in pre-clinical and clinical trials.

The use of natural chemicals in conventional treatments represents a substantial advancement in modern health care. Several natural medicines previously confined to traditional applications are undergoing rigorous effectiveness testing for a variety of medical diseases, as research progresses. This demonstrates a trend toward more holistic and integrative therapeutic methods [40,113]. A meticulous equilibrium between scientific validation and traditional knowledge is necessary for this shift. The clinical efficacy of natural chemicals, such as paclitaxel, highlights the capacity of these substances to supplement, or even improve, traditional medicinal interventions. Nevertheless, the inclusion of these substances in conventional treatment plans is contingent upon stringent clinical validation to ascertain their efficacy and safety when utilized in conjunction with, or in lieu of, synthetic medications. Although natural substances frequently possess low toxicity and tolerance, it is important to perform thorough scientific studies to ascertain their safety and effectiveness in treating a wide range of medical diseases. Clinical trials are vital for identifying the possible advantages and limitations of these natural substances as medical therapies. The continuous process of integrating and conducting comprehensive evaluations indicates an expanding acknowledgment and acceptance of the benefits offered by natural chemicals in contemporary medicine [114,115].

## 5. Computational Approaches in Drug Discovery

Computational methodologies have brought about a paradigm shift in the domain of pharmaceutical discovery, providing indispensable instruments across the entire drug development life cycle. These techniques substantially lower expenses and increase the effectiveness of processes involved in identifying and producing new medications. Prominent computational techniques include docking, virtual high-throughput screening, and protein structure prediction. These methods enable the expeditious evaluation of extensive compound libraries and the detection of potential binders via sophisticated modeling, simulation, and visualization methodologies [116,117]. Various computational drug discovery methods such as molecular docking, pharmacophore modeling and mapping, de novo design, molecular similarity calculations, and sequence-based virtual screening have undergone significant refinement in recent decades. Consequently, the screening and design of drug candidates have become considerably more precise and efficient [118]. The integration of computational techniques into the drug-design and discovery processes has become an essential element of this development, enabling researchers to conserve significant time and money [119]. Furthermore, several computational methods, such as the NMR structure–activity connection, are sophisticated iterations of conventional procedures, illustrating how technology may augment and optimize the process of drug development [116]. As mentioned earlier, developments highlight the pivotal significance of computational approaches in contemporary pharmaceutical research, signifying a substantial paradigm change in the approach to drug discovery and development.

Computational methodologies have become indispensable adjuncts to conventional experimental processes in the realm of cancer drug discovery, substantially bolstering efficacy and diminishing the financial outlay associated with the development of novel therapeutics [117]. The exponential expansion of computational techniques used in the field of drug discovery, particularly those targeted at anticancer medicines, has significantly influenced the design of anticancer drugs. These technologies have yielded significant knowledge on cancer therapy, presenting new opportunities for the discovery and exploration of innovative pharmaceutical candidates [120].

Computational in silico techniques have witnessed significant progress in recent years, particularly in the modeling of biological processes for the identification of novel disease-relevant targets. Significantly contributing to these achievements, machine learning and deep-learning techniques have enabled the identification of novel drug–phenotype and drug–target relationships [121]. The utilization of computational techniques in the development of prospective anticancer medications has yielded significant advancements in the realm of cancer treatment, throughout the years [122]. The development of omics data during the past decade has also enabled computer prediction of anti-cancer therapies, thereby enhancing the efficacy of drug research. For instance, merging high-throughput transcriptome data with drug-response data has been extensively utilized in biomarker identification and medication prediction [123].

Computer-aided drug discovery (CADD) is a promising technology for drug development. This increases the efficiency, effectiveness, and speed of the drug design process, thus tackling the problem of lowering research expenses and accelerating the creation of novel medications. In particular, the influence of CADD in the field of anticancer medicine has revolutionized the design of anticancer drugs and offers essential insights into cancer treatment [120]. In essence, the incorporation of computational methodologies into the realm of cancer drug discovery signifies a fundamental change, presenting novel solutions to conventional obstacles and facilitating the development of cancer therapies that are both more productive and streamlined.

The advent of computational methods for drug discovery has opened new horizons in personalized medicine, particularly in the field of cancer treatment. Computational approaches are increasingly being used to tailor cancer therapies to individual patients, considering their unique genetic makeup and disease characteristics [124]. Through the utilization of computational methods such as genetic data analysis and patient-specific simulation models, scientists can forecast the distinct responses of individual patients to a variety of anticancer medications. Particularly crucial in oncology, where the genetic diversity of malignancies can substantially impact therapeutic results, is an individualized approach. By enabling the identification of particular biomarkers that direct the selection of the most efficacious treatment for each patient, computational approaches eliminate the trial-and-error approach, which is frequently associated with cancer therapy. The transition towards personalized medicine holds the potential to enhance treatment effectiveness and patient outcomes, while concurrently reducing adverse effects and elevating the overall quality of life for patients with cancer. Cancer therapy is being revolutionized by the use of computational technologies in personalized medicine, resulting in enhanced precision, efficacy, and patient-centeredness [125,126].

### 5.1. Molecular Modelling and Drug Design

**Figure 4:**
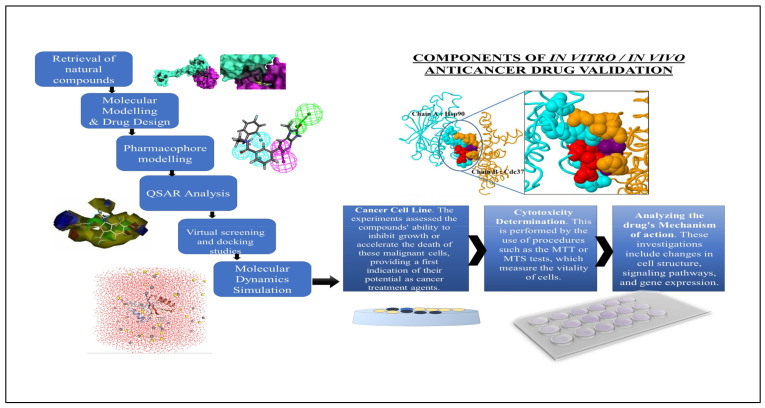
The discovery of natural compounds undergoes various stages, which include computational methods and the validation. The computational methods follow sequential steps that involve retrieval of natural compounds, molecular modelling, pharmacophore modelling, QSAR analysis, virtual screening and molecular dynamic simulation. Based on the binding scores, the top natural compounds against the molecular target will be further validated under the in vitro and in vivo cancer model.

Through the implementation of molecular modeling, computational methods have substantially transformed drug development procedures. This method is implemented by producing, modifying, or expressing three-dimensional structures of molecules and evaluating the physicochemical attributes associated with them. Chemical modeling enables medicinal chemists to predict the molecular and biological characteristics of therapeutic molecules, which is a significant asset in drug design [127,128]. Predicting molecular interactions is an essential component of molecular modeling. This requires modeling the potential interaction between a pharmacological molecule and its target, which may be an enzyme or protein within the body. Medicinal chemists can discern prospective binding sites and predict the characteristics and potency of these interactions through this process. This stage is critical for evaluating the safety and effectiveness of a potential medicine prior to its synthesis and laboratory testing [129,130]. The concept of drug designing via a computational method is illustrated in Figure 4.

Another noteworthy attribute of computational approaches in the realm of drug creation is their capacity to screen extensive collections of molecules rapidly. Complementary to high-throughput screening (HTS) methodologies, computer methods provide rapid evaluation of thousands of molecules. This technique considerably expedites the initial phases of drug discovery, by identifying promising candidates that warrant further research [130]. In addition, computational techniques play an important role in the enhancement of potential pharmaceuticals. By modifying the structure of a molecule using structure–activity relationship (SAR) analysis, chemists are able to augment its medicinal qualities, including, but not limited to, greater potency, decreased toxicity, and enhanced pharmacokinetic profiles. This optimization is essential for the development of safe and efficacious pharmaceutical products [130].

Furthermore, there is a growing trend to employ molecular modeling and computational techniques to predict the pharmacokinetics and pharmacodynamics of potential drugs. These predictions facilitate the comprehension of the physiological actions of medication throughout the human body, encompassing its distribution, excretion, and metabolism. Such data are crucial for determining whether a medicine will be successful in clinical trials [129]. The value and efficacy of molecular modeling in drug development cannot be overstated. This increases the probability of identifying effective and safe medications, provides a more comprehensive understanding of molecular interactions at the atomic level, and decreases the time and expense associated with conventional drug development techniques. Furthermore, with the progress of technology, these computational techniques persistently enhance complexity, facilitating more precise prognostication and streamlined medication-creation procedures [131,132].

An exemplary illustration of its utilization is in the development and refinement of innovative curcumin analogs that hold promise as anticancer medicines. Curcumin, an antioxidant, anticancer, and natural polyphenol derived from the rhizome of Curcuma longa, is well known for its properties. However, its chemical instability and low absorption impede its practical use. To address these obstacles, medicinal chemists have resorted to using molecular modeling methodologies to develop and refine curcumin analogs [128,133].

Molecular modeling methodologies, such as molecular dynamics simulations and docking studies, are crucial for forecasting the binding mechanism and affinity of curcumin analogs towards their respective target proteins. The enhanced binding ability of these improved curcumin analogs for targeting proteins increases their potential as medicines against cancer [133].

Research conducted by Cao et al. exemplifies this methodology. An assortment of innovative mono-carbonyl curcumin derivatives was formulated and manufactured, and their efficacy against hepatocellular carcinoma was assessed via in vitro and in vivo investigations [127]. In anti-proliferation experiments, G2 emerged as the most powerful derivative, relative to curcumin. Additional research employing molecular docking, wound healing, transwell, JC-1 staining, and Western blotting techniques revealed that G2 possesses the ability to impede apoptosis and cell migration by manipulating the expression of apoptosis-related proteins and impeding the phosphorylation of AKT [127]. Additionally, the effectiveness of G2 was verified in a xenograft model using HepG2 cells; H&E staining confirmed that it inhibited tumor development more effectively than curcumin [127].

In summary, molecular modeling is indispensable in the field of drug design. This facilitates the accurate prediction of the chemical and biological characteristics of drug compounds by medicinal chemists. By creating and optimizing new curcumin analogs as possible anticancer medicines, the application of molecular modeling tools demonstrates how this methodology may result in the creation of more powerful medications by improving their binding to target proteins.

#### 5.1.1. Molecular Dynamics Simulations

Molecular dynamics (MD) simulations are crucial for comprehending the temporal behavior of molecules. By simulating the movement of atoms and molecules within a given system, these simulations offer significant insight into the ways in which these components interact dynamically. MD simulations are beneficial in the field of drug design because of their ability to forecast the conformational alterations that occur in a protein when it binds to a drug molecule [134].

Establishing the initial model, which entails delineating the three-dimensional configuration of the molecules under investigation, including possible therapeutic compounds and target proteins, is a critical phase in MD simulations. Frequently, data from experimental methods, such as X-ray crystallography or NMR spectroscopy, are utilized at this stage. Subsequently, the simulation period, temperature, and pressure are adjusted, to replicate physiological conditions. The capability of MD simulations to provide an intricate representation of molecular motion and interactions at the atomic level is a source of power. This is accomplished by employing sophisticated algorithms and computational methods to compute the forces and potential energy between each atom in the system. Through this method, scientists are able to track the dynamic interaction between a molecule, such as a medicine, and its intended target, thereby gaining valuable knowledge of the stability of the molecule, the process of binding, and the possible impacts on the target’s functionality [130,131,135].

Software tools play a crucial role in MD simulation. Programs such as GROMACS, AMBER, and NAMD are widely used because of their robust computational algorithms and ability to handle large and complex molecular systems. These tools require significant computational power, often necessitating the use of high-performance computing clusters or specialized hardware. MD simulations are particularly effective in drug design, for several reasons. They help to identify the most stable binding conformation of a drug to its target, which is crucial for high efficacy. They can also reveal potential off-target effects by simulating how a drug might interact with other proteins, thereby helping to predict and mitigate side effects. Moreover, MD simulations aid in understanding the dynamic nature of proteins, which is often not possible using static experimental methods. Proteins are not rigid structures; they undergo constant conformational changes that can significantly impact their interaction with drugs. MD simulations provide a dynamic view of these changes, offering insights into how a drug might influence a protein’s function in real-time [131,135,136]. The significance of MD simulations in drug research cannot be overstated. Molecular mechanisms are better understood, the rational design of medicinal molecules is guided, and the efficacy and safety of novel therapeutic compounds may be predicted, with their assistance. The ongoing progress in computational power and software capabilities will invariably solidify the significance of MD simulations in the drug discovery process. This will facilitate the development of therapies that are more precisely targeted and efficacious [136].

An important use of molecular dynamics simulations in the field of cancer treatment is the investigation of flavonoid affinity and binding modalities to G-quadruplex DNA. A family of naturally occurring chemicals known as flavonoids has been shown to exert anticancer effects [137]. In cancer treatment, G-quadruplex DNA, which is distinguished by its distinct four-stranded configuration, has surfaced as a prospective target, because of its substantial influence on gene-expression regulation [138].

Using MD simulations, scientists have examined the interaction between flavonoids and G-quadruplex DNA. The use of these models is crucial in forecasting the affinity of flavonoids for binding to G-quadruplex DNA, which is a pivotal determinant in evaluating their potential as medicines against cancer [139,140]. The structure of flavonoids, including their hydrophobicity and planarity, as well as the presence and location of hydroxyl groups on the flavonoid molecule, affect their binding affinity.

Zhang et al. conducted a noteworthy investigation by employing molecular dynamics simulations to examine the affinities and mechanisms of flavonoid binding to G-quadruplex DNA. According to the results obtained from this study, flavonoids characterized by a hydrophobic surface and a planar structure exhibited a higher propensity to bind efficiently to G-quadruplex DNA [141]. Furthermore, the research findings revealed that the quantity and placement of hydroxyl groups on flavonoids influenced the binding affinity. This underscores the critical role that molecular structure plays in the effectiveness of binding [140,141].

In essence, molecular dynamics simulations play a crucial role in the domain of drug design by facilitating the anticipation of protein responses to bind drug-molecule. Through investigation of the binding affinities and mechanisms of flavonoids to G-quadruplex DNA, these simulations provide a substantial contribution to the body of knowledge about the possible anticancer properties of flavonoids. The insights gleaned from these simulations guide the creation and design of more efficacious medicines for cancer.

#### 5.1.2. Virtual Screening and Docking Studies

Docking studies and virtual screening are the fundamental computational methods used in drug development. By utilizing computer algorithms, virtual-screening sites among vast databases of chemicals can be used to discover those that have a significant probability of binding to a target protein [142]. Docking studies offer a supplementary perspective, by forecasting the method by which a small molecule can connect with a target protein, thereby revealing information regarding a compound’s potential efficacy and interaction dynamics [143,144].

The virtual-screening procedure comprises several essential stages. The process begins with the identification of a target, often a protein, linked to a certain ailment; following this, a search is conducted across databases with millions of chemical structures, to discover the molecules most likely to bind to the chosen target. The algorithms utilized in this procedure assess the structural compatibility and physicochemical features of compounds with the target protein. Sophisticated software applications, such as AutoDock, GOLD, and Schrodinger suite tool Glide, are frequently used to achieve this objective. These techniques rank compounds according to their expected binding affinities using scoring systems, enabling scientists to concentrate on the most promising possibilities [129,145]. Understanding the manner in which the structural characteristics of a potential medicine affect its interaction with the intended biological target and information that is critical for maximizing pharmacological efficacy are highlighted within these investigations comments [116,129].

The effectiveness of these computational approaches lies in their capacity to efficiently and economically evaluate extensive collections of chemicals, thereby substantially reducing the number of prospective therapeutic candidates that require additional experimental investigation. This not only expedites the process of drug development, but also diminishes the necessary resources and duration of laboratory and clinical trials. Virtual screening and docking studies play a crucial role, not only in the identification of new drug candidates, but also throughout the lead optimization phase of the drug development process. Therapeutic chemists are guided in their efforts to enhance the effectiveness, selectivity, and pharmacokinetic features of lead compounds by means of these techniques, which provide comprehensive knowledge of the molecular interactions between the drug and its target [116]. Moreover, the accuracy and dependability of these computational methods are improving, as algorithms, software, and processing power continue to increase. This advancement is improving the accuracy with which drug–target interactions can be predicted, which is a critical factor in the advancement of safer and more effective pharmaceuticals.

An exemplary implementation of these methodologies is demonstrated by a case study pertaining to the virtual screening of a database of natural marine materials. Inhibitors of the STAT3 signaling system, a crucial regulatory route for cell survival and development that is frequently dysregulated in several forms of cancer, were the focus of this investigation [142,146].

Using molecular docking techniques, the virtual-screening campaign was conducted using a database containing more than 90,000 natural products and natural product-like compounds. The objective was to identify specific chemicals that might impede the STAT3 signaling pathway. A comprehensive screening procedure resulted in the identification of fourteen hit compounds, with “compound 1” emerging as the most promising contender [147].

The potential of compound 1 was demonstrated by the in vitro inhibition of STAT3 DNA-binding activity. Furthermore, it demonstrated efficacy in suppressing transcription directed towards STAT3 in the cell, demonstrating selectivity in comparison to STAT1. Significantly, its efficacy was comparable to that of the widely recognized STAT3 inhibitor, S3I-201. The discovery has notable importance, as it underscores the possibility of “compound 1” functioning as a feasible substitute for presently available STAT3 inhibitors [147].

Virtual screening and docking studies provide medicinal chemists with an effective method for identifying prospective drug candidates, rendering them indispensable instruments in the field of drug development. A practical application of these methodologies is illustrated by a case study of screening natural marine materials for STAT3 inhibitors. This demonstrates the effectiveness of virtual screening and docking experiments in identifying ‘compound 1,’ a highly promising candidate that has similar effectiveness to well-established STAT3 inhibitors, such as S3I-201.

#### 5.1.3. Pharmacophore Mapping and QSAR Analysis

Pharmacophore mapping and quantitative structure–activity relationship (QSAR) analysis are crucial computational methods in the realm of drug development. Pharmacophore mapping identifies the essential structural components of a compound that govern its biological activity. In contrast, QSAR analysis entails the use of statistical models to forecast the biological activity of a compound, according to its chemical structure [148,149].

Quantitative structure–activity relationship (QSAR) analysis and pharmacophore mapping are fundamental computational methods utilized in the domain of pharmaceutical innovation. The objective of pharmacophore mapping is to discern the essential structural characteristics of a drug that are critical for its physiological function. These characteristics may consist of, among others, donors or acceptors of hydrogen bonds, hydrophobic areas, and aromatic rings. The discernment of these characteristics facilitates the comprehension of the manner in which various chemicals engage with biological targets, such as enzymes or receptors [116,150].

In contrast, QSAR analysis predicts the biological activity of a substance based on its chemical structure, using statistical models. This strategy involves linking the physicochemical attributes and observable biological activities of a series of chemicals. A mathematical model is constructed by analyzing parameters such as molecular weight, lipophilicity, electronic properties, and steric factors to forecast the activity of novel compounds [150,151]. Pharmacophore mapping frequently starts with the examination of a collection of drugs that are recognized to interact with the target of interest. Sophisticated software applications, such as Discovery Studio (version 2.13), Molecular Operating Environment (MOE) (version 2022.02), and Schrodinger (version 2023-4), are employed to discern shared structural characteristics among these bioactive molecules. The pharmacophore model is constructed around these common characteristics, and may then be employed to filter chemical libraries in the search for novel molecules harboring these crucial attributes [150,152]. The initial stage of QSAR analysis typically entails the compilation of a dataset, including chemicals with established biological activities. A multitude of descriptors were computed to symbolize the chemical characteristics of these molecules. Statistical tools, including machine learning and regression analysis, were subsequently utilized to construct a model that established a relationship between biological activity and the descriptors. Software applications such as SYBYL-X, Open3DQSAR, and ChemOffice are frequently used [153].

The capacity of pharmacophore mapping and QSAR analysis to direct the creation of novel compounds with targeted biological activity is crucial for the drug development process. By comprehending the fundamental characteristics necessary for activity and the correlation between structure and activity, these approaches have the potential to substantially diminish the temporal and resource investments necessary to identify prospective pharmaceutical candidates [153]. Moreover, these methodologies are crucial throughout the lead optimization phase. Alterations to the chemical configuration of a lead product can be proposed, to improve its pharmacokinetic characteristics, selectivity, and potency. For the development of a medicine that is not only effective, but also safe, for human use, this optimization procedure is crucial. Continuous progress in processing capacity, software advances, and algorithmic sophistication have significantly improved the precision and dependability of pharmacophore mapping and QSAR analysis. With ongoing advancements in these technologies, they are progressively assuming a critical role in the process of drug discovery and development. This integration will facilitate the expeditious and effective creation of novel therapeutic agents [154,155].

The creation of a pharmacophore model for inhibitors of histone deacetylase (HDAC), an enzyme target of considerable importance in cancer research, is an outstanding case study in this field. Regulation of gene expression is a critical function of HDACs, which are frequently dysregulated in various malignancies [156,157].

Utilizing a library of natural compounds and structure-based pharmacophore modeling, in conjunction with proven QSAR analysis, this study sought to identify new HDAC inhibitors [158,159]. By integrating molecular descriptors and a structure-based pharmacophore, the QSAR model utilized in this investigation adequately elucidated the variability in the bioactivity of structurally distinct HDAC inhibitors [160,161]. The effectiveness of the pharmacophore model was verified by the utilization of a receiver operating characteristic curve; further analysis opted for the QSAR equation that was deemed to be statistically optimal [160]. This model was then employed as a 3D search query, to determine the AnalytiCon Discovery database for natural products [161].

This work led to the discovery of novel chemical scaffolds that function as HDAC inhibitors. These scaffolds offer encouraging initial steps toward optimizing the structure of leads. The efficacy of pharmacophore mapping and QSAR analysis in identifying prospective drug candidates, specifically within the domain of cancer therapies, was highlighted in this case study [160,161].

Pharmacophore mapping and QSAR analysis are crucial instruments in the realm of drug development, enabling medicinal chemists to effectively identify and refine prospective drug candidates. By creating a pharmacophore model for HDAC inhibitors obtained from natural products, these methodologies can be utilized to identify novel chemical scaffolds, thereby facilitating the development of innovative cancer therapies.

#### 5.1.4. From Computational Studies to the Bench: Experimental Validation

A critical juncture in the process of drug discovery occurs when computational investigations are succeeded by experimental validation, specifically with anticancer medicines generated from natural chemicals [40]. This pivotal stage guarantees that the encouraging outcomes derived from computer models can be successfully translated into practical therapeutic applications.

A significant point in the drug discovery process is the move from computational research to experimental validation, particularly in the creation of anticancer medications derived from natural substances. This critical stage guarantees the successful conversion of encouraging outcomes derived from computer models into tangible treatment implementations, thereby bridging the gap between theoretical forecasts and actual effectiveness. Upon the identification of promising drug candidates through computational approaches, such as molecular docking, virtual screening, QSAR analysis, and pharmacophore mapping, experimental validation was conducted, by subjecting these findings to a battery of stringent testing. Typically, this procedure begins with in vitro investigations, during which the biological activity of drugs is assessed, using cell cultures. These investigations contributed to the preliminary validation of the safety and effectiveness of these compounds at the cellular level [162,163].

When drugs demonstrate efficacy in vitro, they are frequently subjected to in vivo investigation, using animal models. The purpose of these investigations was to evaluate the pharmacokinetics of the compounds (the manner in which the medication is absorbed, distributed, metabolized, and eliminated inside a living body), and the pharmacodynamics (the biological effects of the drug on the organism). Additionally, they assessed possible adverse effects and toxicity, which are critical factors in evaluating a drug’s safety profile.

During these experimental phases, a variety of spectroscopy, chromatography, and mass-spectrometry methods were implemented, to identify the compounds and determine their purity. Enzyme inhibition tests and other biochemical assays were performed, to determine the biological activities of the substances. In vivo investigations employ sophisticated imaging modalities such as MRI and PET emission tomography, to observe the patterns of drug distribution and assess their physiological impacts. The effectiveness of this progression from the computational to experimental phases is critical for successful drug development. Although computational approaches can screen and optimize large quantities of molecules in a timely and economical manner, their predictions must be empirically confirmed, to verify their relevance in the real world. In the development of anticancer medications, where the intricacy of cancer biology frequently necessitates exhaustive experimental testing to establish the efficacy and safety of novel therapies, this validation is particularly crucial [164,165,166].

Moreover, this experimental validation procedure is in a constant state of evolution, as technological and methodological developments improve the precision and efficacy of these examinations. For example, the utilization of high-throughput screening technology has substantially expedited the process of in vitro testing, by enabling the simultaneous testing of thousands of compounds.

## 6. In Vitro Assays

Effectively reconciling computational predictions with experimental validation is of utmost importance in the complex endeavors of anticancer drug development. Based on their anticipated interactions with cancer-associated biological targets, computational investigations have employed sophisticated simulations and virtual screens to identify prospective therapeutic candidates. Experimental validation, specifically in vitro tests, is the means by which the true efficacy and safety of these candidates can be ascertained [167,168]. The utilization of these tests is critical for validating whether the hypothesized anticancer effects of drugs are manifested in actual biological responses.

Understanding this process is of utmost importance, and we shall investigate it by means of a concentrated case study: the scrutiny of betulinic acid, a molecule whose anticancer potential was computationally determined, and its verification, using in vitro testing against melanoma cell lines [169,170]. Effectively connecting computational predictions with experimental validation is of the utmost importance in the complex process of anticancer medication development. Computational investigations employ sophisticated simulations and virtual screens to discern prospective treatment contenders, by analyzing their anticipated interactions with biological targets associated with cancer. The final assessment of the candidates’ safety and effectiveness was conducted through experimental validation, with a specific focus on in vitro experiments. These assays are critical in verifying whether the postulated anticancer properties of pharmaceuticals are demonstrated by tangible physiological reactions [164,171].

A range of approaches and procedures are used in in vitro tests to determine the biological activity of substances in a controlled laboratory setting. Typically, cancer cell lines, which are cells obtained from a variety of tumor types, are utilized in the execution of these procedures. The experiments evaluate the capacity of the compounds to impede proliferation or stimulate the death of these malignant cells, offering a first indication of their potential as therapeutic agents against cancer. An essential component of in vitro experiments is the quantification of the medication dose necessary to eradicate a specific proportion of cancer cells, a process known as cytotoxicity determination. This is often accomplished using methods such as the MTT or MTS tests, which quantify the vitality of cells. Another critical element involves evaluating the mechanisms of action of chemicals, including whether they promote programmed cell death (apoptosis) or impede specific pathways that are essential for the survival of cancer cells [172,173,174].

The experiments employed a variety of tools and equipment, ranging from rudimentary laboratory instruments such as pipettes and microscopes to more advanced devices such as high-content screening systems and flow cytometers. These technologies facilitate comprehensive scientific studies on the impact of chemicals on cancer cells. Such analyses encompass alterations in gene expression, signaling pathways, and cell shape. The impact of the software on the analysis of data acquired from these tests is substantial. The biological implications of the chemical interactions with cancer cells were deduced [175,176,177].

The capacity of in vitro tests to efficiently and economically screen a substantial quantity of compounds contributes to their effectiveness in drug development. The insights provided by these tests into the possible therapeutic effects of novel drugs will serve as a guide for subsequent research and development. It is essential to remember, however, that although in vitro tests are a crucial component of drug development, they are only the beginning of a lengthy process. Subsequent to these screens, compounds that exhibit potential necessitate additional validation, through in vivo investigations and clinical trials, to comprehensively ascertain their safety and effectiveness in living organisms [172,173].

As a possible anticancer drug, betulinic acid, a naturally occurring triterpenoid, was identified using computational approaches. The determination of this entity was predicated on its anticipated interactions with. and efficacy against, certain targets associated with melanoma, a grave type of skin cancer [178,179]. Molecular docking was performed on the binding sites of potential anticancer targets, including topoisomerase I and II, epidermal growth factor receptor (EGFR), vascular endothelial growth factor (VEGFR), transcription factor NF-κB, anti-apoptotic protein Bcl-2, and peroxisome proliferator-activated receptor (PPARγ) [180,181]. According to the docking results, the best fit to the PPARγ binding pocket was shown by the compound-4 (3-diethoxyphosphoryl-28-propynoylbetulin), which is a derivative of betulin [182]. For experimental validation, betulinic acid was subjected to a series of in vitro experiments, using various melanoma cell lines. To faithfully reproduce the conditions that govern computer predictions, this approach was carefully crafted. The tasks included in this study were to ascertain the optimal doses and times of betulinic acid exposure, in order to evaluate its anticancer properties. The objectives of the experiments were to quantify a range of characteristics, including apoptosis induction, cell viability, and molecular markers, which serve as indicators of anticancer action [183,184,185].

These findings demonstrated that the viability and confluence of every examined cell line decreased in a dose-dependent manner. In contrast, SK-MEL-5 cells were more susceptible to betulinic acid than B16-F10 cells, suggesting that healthy, melanoma-free SK-MEL-5 cells and pigmented B16-F10 cells have distinct cytotoxic effects [185,186]. Further studies are required to confirm the interaction between betulinic acid and melanin. The experimental confirmation that betulinic acid has anticancer effects against melanoma cell lines highlights the significance of in vitro testing throughout the pipeline of drug discovery. This case study serves as a prime illustration of the collaborative potential of experimental research and computational predictions, emphasizing the significance of in vitro tests in validating and broadening our understanding of prospective anticancer drugs. Additionally, the results establish a foundation for subsequent investigations, such as in vivo trials, to thoroughly examine the therapeutic capabilities of betulinic acid in melanoma treatment. This converts computational forecasts into practical clinical applications [185,186].

## 7. In Vivo Studies

These studies encompass the evaluation of prospective anticancer substances in live organisms, predominantly animal models. This study aimed to assess the overall biological and pharmacological effects of chemicals within a complex biological system, encompassing toxicity, metabolism, and therapeutic effectiveness. In vivo studies are important for validating the anticancer properties identified in vitro and for determining the generalizability of a molecule as a therapeutic agent. Important information on dose, adverse effects, and pharmacokinetics is also provided by these studies; this information is crucial for the progression of drug candidates to clinical trials [187,188].

In the key phase of anticancer drug research, in vivo studies have examined potential therapeutic chemicals in living organisms, mostly animal models. The purpose of these investigations was to evaluate the pharmacological and biological effects of chemicals on a complex biological system. Critical elements, such as toxicity, metabolism, and therapeutic efficacy, have been thoroughly examined [189,190]. Numerous critical phases are involved in in vivo research procedures. At the outset, an appropriate animal model that closely emulates the physiological and pathological circumstances of the human cancer under investigation was chosen. Mouse, rat, and occasionally rabbit models are frequently employed, with the selection process contingent upon the specific cancer type and molecular processes implicated [191].

After establishing the animal model, chemicals were supplied, and a multitude of parameters were observed. These encompass the efficacy of the drug in diminishing tumor dimensions or impeding its progression, its influence on the animal’s general well-being and conduct, and any indications of toxicity or detrimental consequences. Detailed pharmacokinetic studies, which assess the absorption, distribution, metabolism, and excretion of the drug in the live body, were also conducted during this phase. These studies provided vital data on the optimal dose, possible adverse effects, and overall safety profile of the treatment [189]. The tools and methodologies employed for in vivo investigations are multifaceted and advanced. To monitor tumor growth and medication distribution in the body, they employ imaging technologies like MRI and PET scans, in addition to biochemical tests that quantify drug concentrations and metabolic alterations. Histopathological studies, which involve the microscopic examination of tissue samples, are frequently used to evaluate the cellular and molecular effects of treatment on cancer cells and adjacent tissues [192]. The use of software for data processing in in vivo research is crucial. The importance of the data was determined using statistical tools, and the results were analyzed, in addition to image analysis and simulation of the pharmacokinetics and pharmacodynamics of the medication; specialized software can be employed in these applications [171].

It is impossible to emphasize enough the significance of in vivo investigations in the discovery of anticancer drugs. They provide crucial data that are not attainable through in vitro investigations, particularly with regard to the drug’s action when included in an entire organism. This includes knowledge of potential immunological reactions, drug efficacy in penetrating the tumor at optimal doses, and its overall influence on the health of the organism [190]. Bridging the gap between laboratory research and clinical trials requires in vivo studies. The information gathered from these investigations is crucial for the development of human clinical trials and regulatory authorization. They guarantee that only the most viable and secure pharmaceutical candidates advance to further phases of research, resulting in the provision to patients of safe and efficacious anticancer therapies [189].

The anticancer potential of Withaferin A, a chemical derived from the plant *Withania somnifera*, was evaluated in mouse models of breast cancer. In vivo investigations constitute the subsequent phase in the experimental validation procedure of anticancer medication discovery, subsequent to pivotal in vitro experiments. Conducting these investigations is crucial for assessing the safety and therapeutic effectiveness of prospective pharmaceutical candidates inside a live organism, thereby facilitating a thorough understanding of their pharmacokinetics and pharmacodynamics [193].

The potential anticancer activities of Withaferin A (WFA), a bioactive molecule derived from the herb *Withania somnifera* (often referred to as Ashwagandha), have been uncovered by computational research. Previous investigations, which encompassed molecular docking and simulations, postulated that Withaferin A may efficaciously target critical pathways implicated in the progression of breast cancer. The antitumor potential of WFA against molecular targets that preserve the stemness of breast cancer stem cells was investigated [194,195]. The in vivo investigations revealed a substantial inhibition of tumor development in mice models that were administered Withaferin A. The significant reduction in tumor size and postponement of tumor growth was observed in comparison to the control group. Furthermore, at therapeutic levels, WFA has a favorable safety profile, with few reported side effects [196].

Furthermore, the findings provide insights into the possible processes by which Withaferin A operates. This chemical reduces angiogenesis and promotes apoptosis in cancer cells, both of which are essential steps in the formation and progression of cancer. Withaferin A interactions with various molecular targets, including NF-κB, STAT, Hsp90, ER-α, p53, and TGF-β, have been noted to inhibit cancer cell proliferation and induce cell cycle arrest in the G2/M stage, ultimately leading to apoptosis or cell death.

By promoting programmed cell death, WFA inhibited the growth of MDA-MB 231 cells, a subtype of breast cancer cells, in trials with female nude mice. An alternative study assessed the anticancer properties of WFA in relation to breast cancer. This is achieved by impeding autophagic flow, obstructing lysosomal activity, and stimulating apoptosis via energy impairment [193,194,195,197].

The potential of WFA as a therapeutic agent has been validated by in vivo investigations, underscoring the criticality of conducting clinical trials to assess its efficacy in the treatment of human breast cancer. This underscores the need to integrate computer forecasts into practical clinical settings, to establish a connection between laboratory investigations and the provision of healthcare for patients.

## 8. Clinical Trials: The Journey to Therapeutics

The process of anticancer drug development, which starts with the transformation of natural products from laboratory studies to clinical use, is complex and vital. This process involves preclinical investigations and subsequent clinical trials, with each stage being critical for the conversion of natural substances into effective treatments [58].

Clinical trials signify the ultimate, and possibly most pivotal, phase in the development of anticancer medication, wherein undiscovered substances are converted from laboratory experiments to practical therapeutic uses. The comprehensive progression of this procedure, including preclinical inquiries and subsequent clinical trials, is critical for the precise conversion of natural compounds into efficacious and risk-free cancer medicines. Preclinical research, encompassing in vivo studies and in vitro tests, establishes a foundation by providing critical knowledge about the safety, effectiveness, and pharmacological characteristics of a potential medicine. However, these compounds have been evaluated in humans during the clinical trial phase, to determine their genuine medicinal potential and safety profile [198,199].

### 8.1. Preclinical Studies

Preclinical investigations mark the crucial first step in advancing potential drugs from discovery to human trials. They are essential for understanding the safety and efficacy of compounds identified through computational research and in vitro and in vivo studies. Their primary aim is to evaluate safety through toxicological assessments, determining safe dosage ranges and pinpointing potential side effects. Additionally, these studies involve assessing effectiveness, often using animal models resembling human cancer scenarios, to gauge the potential therapeutic advantages of these compounds [200,201].

Moreover, preclinical investigations involve thorough ADME profiling (Absorption, Distribution, Metabolism, and Excretion), to gain a more profound insight into how these compounds behave within biological systems. This comprehension is vital for predicting their behavior in human subjects, particularly concerning how they are absorbed, distributed, metabolized, and excreted, as well as their availability in the body and metabolic paths. Studying pharmacokinetics and pharmacodynamics is of significant importance, as it explores how the compound moves through the body and interacts with biological targets. This understanding is pivotal for improving methods of formulation and delivery [202,203]. To enhance the comprehension of the extent and importance of preclinical investigations, a particular case study was carried out to assess possible anticancer capabilities of Silvestrol, a chemical obtained from *Agnostemma silvestre* (alternatively referred to as Aglaia foveolata). The pharmacokinetics and anticancer properties of silvestrol were assessed in mice. According to this study, it displays strong anticancer activity against several cancer cell lines, including breast, prostate, and pancreatic tumors. Additionally, the drug exhibited acceptable pharmacokinetic characteristics, and was well tolerated by animals [204]. Its mechanism of action is a primary focus of preclinical research, where it is observed that it inhibits eukaryotic initiation factor 4A (eIF4A), crucial for protein synthesis in cancer cells. The disruption of critical mRNA translation mechanisms caused by this inhibition hinders the proliferation and viability of cancer cells [204,205].

As exemplified by Silvestrol derived from Agnostemma silvestre, the progression of developing anticancer drugs from natural compounds to clinical application underscores the crucial role of preclinical studies in connecting laboratory-based research. These studies serve a dual purpose, offering a comprehensive understanding of the safety and effectiveness of the compound, while establishing the groundwork for its transition to clinical trials. The potent anticancer properties and pharmacokinetics revealed through preclinical investigations provide a solid foundation for subsequent phases of pharmaceutical development. The incorporation of advanced technologies like next-generation sequencing and high-throughput screening represents significant advancements, streamlining and refining the assessment process. These state-of-the-art tools enhance the precision of preclinical investigations and open avenues for innovative approaches to personalized medicine within the realm of cancer therapy [198,204]. Through the systematic and thorough application of this approach, coupled with the integration of technological progress, it becomes possible to exclusively authorize clinical trials for the most promising and safe pharmaceutical candidates. This comprehensive strategy not only accelerates the drug discovery process but also notably enhances cancer therapy and increases patient well-being.

### 8.2. Clinical Trials

After completing preclinical investigations, the pharmaceutical discovery process moves into clinical trials. This pivotal phase involves testing potential medications on human participants, building upon earlier in vitro and in vivo assessments. Clinical trials are essential for establishing the safety and effectiveness of medicines for treating different illnesses in diverse human populations. These trials are systematically divided into phases, and meticulously assess the medication’s therapeutic potential, optimal dosage, side effects, and overall effectiveness when compared to standard treatments [206].

Consisting of four different phases, each stage of a clinical study is characterized by unique aims and progressively greater levels of participant engagement. In Phase I trials, new medications are evaluated in a limited population to determine the safest dose range and to discover adverse effects. Phase II studies, which are carried out on more extensive cohorts, evaluate the efficacy of therapies that have been deemed safe in Phase I. Involving even bigger cohorts, the objectives of Phase III studies are to validate the efficacy of the treatment and monitor adverse effects. Phase IV clinical studies are conducted following the regulatory approval of the treatment, and are designed to assess its efficacy and long-term safety [206,207].

Artemisinin, which is extracted from the sweet wormwood plant (*Artemisia annua*), is widely recognized as an antimalarial medication. Recent studies have highlighted the potential of artemisinin as a treatment for various cancers. To explore this potential, clinical researchers conducted an assessment of artemisinin, revealing its ability to destroy cancer cells in leukemia, colon, breast, lung, and pancreatic cancers. It is important to note that these promising findings stemmed from initial investigations conducted on animal models; however, more rigorous clinical trials involving human subjects are necessary to substantiate these assertions. Artemisinin evolution from a traditional antimalarial drug to a potential cancer treatment stands as a significant example of the path natural compounds take in drug development. The phases of clinical trials for artemisinin were as follows [208,209].

Phase I trials assessed artemisinin’s safety and tolerability in humans, involving a small number of healthy volunteers or patients with the disease. The main goals were to identify immediate adverse effects and establish a safe dosage range. Phase II trials, aimed at gauging artemisinin’s effectiveness as a cancer treatment, and involved a larger group of patients. The focus was on evaluating the drug’s anticancer properties, while continuously monitoring any adverse effects. Often, these studies were randomized, and included control groups. Phase III trials were conducted to confirm artemisinin’s effectiveness. These trials compared artemisinin against established standard cancer treatments, gathering extensive data for drug approval, and monitoring adverse effects.

Furthermore, Krishna et al. conducted a randomized, double-blind, placebo-controlled experiment that assessed the efficacy of oral artemisinin (ARS) in the treatment of colorectal cancer (CRC). The anti-proliferative properties and general tolerability of ARS in colorectal cancer (CRC) were demonstrated in a trial, suggesting its potential as a cancer treatment agent [210].

#### Integration of Biomarkers and Personalized Medicine in Clinical Trials

The incorporation of biomarkers and personalized medicine into clinical trials represents significant advancement in evaluating natural compounds like artemisinin for cancer treatment. Biomarkers, found in tissues, blood, or bodily fluids, are increasingly used to predict and monitor how cancer responds to treatment. Leveraging specific biomarkers in artemisinin clinical trials could provide vital insights into how the drug works and its effectiveness against different types of cancer. Personalized medicine, adjusting therapy based on individual patient traits, is gaining importance in clinical trials [211].

This approach strives to create customized treatment plans that are more effective, by considering genetic, environmental, and lifestyle factors. By integrating personalized medical approaches and studying biomarkers in clinical trials, researchers can gain deeper insights into how natural products interact with different molecular and genetic cancer characteristics. This leads to the creation of more precise and efficient treatment plans. Shifting towards a more personalized approach in clinical trials not only increases the likelihood of successful treatments, but also significantly decreases potential side effects, making a substantial impact on advancing cancer therapy [212]. Clinical trials of artemisinin in cancer therapy underscore the potential of natural chemicals in the advancement of novel therapeutic agents. They underscore the significance of conducting thorough assessments to ascertain the safety and effectiveness of these chemicals in the treatment of human ailments. The transformation of artemisinin from its traditional use to its clinical application in cancer treatment exemplifies the immense medical potential of natural products, which may provide patients with more effective and diverse treatment options, and potentially pave the way for novel approaches to cancer treatment.

## 9. Challenges and Limitations

Discovering anti-cancer medications from natural sources faces numerous hurdles, which slow down the development of effective treatments. The intricate molecular compositions of these natural products pose significant challenges, due to their complexity, making it difficult and resource-intensive to separate, identify, and synthesize them [213]. Moreover, the efficacy of natural compounds as anti-cancer drugs is hindered by limited absorption and distribution in the body, leading to concerns about their bioavailability [214]. Toxicity is a critical consideration, since certain natural substances can pose potential risks, necessitating thorough toxicological studies to ensure patient safety. Additionally, resistance to treatments derived from natural products may emerge in cancer cells, over time. Therefore, a deeper understanding of resistance mechanisms and the development of new strategies to counteract this resistance are essential.

Sourcing natural substances also creates sustainability challenges, as unsustainable farming practices and overharvesting can harm ecosystems and exhaust these resources [215]. The case study of Combretastatin A4, derived from African bushwillow, exemplifies these concerns. Its low solubility and instability in water present significant obstacles to its clinical development. Researchers have tackled these issues by developing a water-soluble version, Combretastatin A-4 phosphate (CA-4P), and prodrug forms to enhance delivery and optimize therapeutic efficacy. Concurrently, employing computer models for natural-product-based drug development faces obstacles, due to the intricate and diverse structures of these molecules [130]. Scarce experimental data, limited databases, and high structural flexibility further impede precise modeling. Variations in chemical makeup and bioactivity, along with poorly understood mechanisms of action, complicate the creation of standardized models [215]. Challenges such as overfitting, substantial computational demands, and the necessity for rigorous experimental validation by regulatory bodies further add to these complexities.

Progress in utilizing natural ingredients for anticancer treatments faces major setbacks, due to intellectual property and patent issues. Often, complex legal matters surrounding the patenting of biological materials complicate the process for securing patents on molecules derived from living organisms. Biopiracy, which arises when indigenous groups are not fairly compensated for their traditional knowledge, presents another barrier. Legal and financial hurdles can impede research and development, alongside ethical concerns raised by these challenges. Striking a balance between protecting the rights of corporations and researchers, while acknowledging the valuable contributions and knowledge of traditional communities, is vital in addressing these intellectual property concerns [216,217].

The recruitment of participants and the design of clinical trials provide an additional set of obstacles in the development of anticancer medicines derived from natural products. The complexity of designing studies that assess the safety and effectiveness of these drugs stems from their varied characteristics. Recruiting an adequate number of volunteers who satisfy the precise criteria for these studies might pose challenges, especially in the case of rare or severe diseases. In light of these obstacles, novel trial-design methodologies, including adaptive trials and efficient patient recruitment and retention tactics, are required, to guarantee that clinical investigations provide dependable and significant findings [218,219].

### Strategies to Overcome the Challenges

The relevance of ancient knowledge is increasingly being acknowledged, with the development of anticancer drugs derived from natural ingredients. Innovative medicinal compounds may be developed with the assistance of indigenous people and healers who have utilized these natural medicines for millennia. By adopting this strategy, researchers not only gain access to an abundance of untapped therapeutic chemicals and plants, but also promote a drug development process that is more inclusive and ethical. The establishment of collaborative alliances that demonstrate reverence and recognition of traditional wisdom may facilitate the identification of innovative anticancer drugs and save this priceless information for posterity [220,221]. Advanced analytical methods such as mass spectrometry and nuclear magnetic resonance have greatly boosted the characterization of complex natural-product structures, leading to greater knowledge of their chemical characteristics and medicinal potential [222].

Nanotechnology plays a significant role in enhancing the bioavailability of natural products. Nanoparticle-based medication carriers have the potential to increase the solubility and stability of these chemicals, allowing better absorption and distribution inside the body [223]. Furthermore, studying combination treatments that incorporate natural products and conventional anticancer medications might produce synergistic results. This method can assist in overcoming resistance mechanisms and enhancing treatment effects, by concurrently addressing several routes [223].

Reducing the toxicity of natural product-derived chemicals is another key concern. Strategies could entail prodrug development, where less hazardous precursor molecules are transformed into their active forms within the body, or devise focused drug-delivery methods to limit off-target effects. In addition, fostering sustainable techniques for gathering and cultivating natural products is crucial for safeguarding ecosystems and guaranteeing a steady supply of these valuable substances. Thus, ethical and ecologically acceptable procurement strategies are vital [224].

The combination of artificial intelligence (AI) and machine learning in this sector is set to transform the discovery and prediction of prospective natural-product-based anticancer medicines. AI systems can evaluate large datasets to locate molecules with high therapeutic potential, thereby accelerating the drug discovery process. Moreover, substantial clinical trials are essential to evaluate the safety and effectiveness of natural-product-derived anticancer medicines in humans. These studies are critical for providing the evidence needed to bring promising drugs from the laboratory to clinical practice [225]. Recent developments in deep learning have substantially altered drug discovery processes. Researchers have employed deep neural networks to study and predict drug-related parameters, including the bioactivity of prospective anticancer drugs. The future of drug development, especially in the field of natural products, entails combining deep-learning algorithms with standard computer methodologies. This technique is intended to boost the accuracy of predictions of the anticancer potential of natural chemicals [226].

A case study in this area involves the prediction of anticancer potential using deep learning combined with standard computational approaches. This methodology combines the use of deep learning for pattern identification and data extrapolation in large datasets of natural chemicals, with molecular modeling and bioactivity predictions from established computational approaches. The intended effect is an improvement in the accuracy of identifying interesting anticancer chemicals from natural sources, leading to a more efficient drug discovery method [225,226]. Furthermore, the use of advanced computational technologies, such as quantum computing and sophisticated software, to model molecular interactions is expected to further refine this integrated approach, paving the way for groundbreaking discoveries in the field of anticancer drug development from natural products [225].

Further advancement in public–private collaborations represents a significant prospective trajectory in the domain of developing anticancer drugs derived from natural products. By combining their resources, technology, and experience, academic institutions, government agencies, and pharmaceutical corporations can overcome the obstacles in this sector, through collaboration. These collaborations can promote the exchange of information, eliminate redundant endeavors, and guarantee that the medication development process operates with optimal efficiency and efficacy. Collaboration between public and private institutions can expedite the process of translating research discoveries into effective cancer therapies, contributing to the improvement of patients and the progression of the oncology field [205,227,228,229].

## 10. Conclusions and Future Direction

The exploration of natural products as potential anticancer drugs represents a significant milestone in medical research, blending modern scientific approaches with the time-tested principles of traditional medicine. Examining historical breakthroughs, like the development of groundbreaking medications such as Taxol, Vincristine, and Vinblastine, underscores the enduring efficacy of natural products. Beyond highlighting the therapeutic potential of natural remedies, these case studies underscore the complex journey of drug development. Concurrently, the field has undergone a transformation, with the integration of computer methods such as molecular modeling, dynamics simulations, and virtual screening. Advances in these technologies have empowered researchers to investigate the interactions of flavonoids with cancer targets and optimize curcumin analogs. This progress facilitates the identification and enhancement of naturally occurring compounds, bridging the divide between theoretical research and practical applications.

Despite notable strides in developing anticancer drugs from natural sources, substantial challenges persist. These challenges encompass issues related to solubility, formulation, and resistance, which have surfaced in the advancement of anticancer drugs. These hurdles underscore the vital need for ongoing innovation and adaptability within the field. Looking ahead, there is considerable potential for merging advanced computer techniques, such as deep learning, with conventional drug development processes. The integration of cutting-edge technologies and well-established methodologies is envisioned to enhance the precision and efficacy of future treatments. The arena of creating anticancer drugs from natural products not only showcases the fusion of traditional wisdom and scientific advancements, but also represents a dynamic frontier in medical research. This resurgence instills optimism for effective cancer treatments, and lays the groundwork for upcoming breakthroughs in healthcare.

## Data Availability

This study did not report any data.
